# Acute onset anterior uveitis after darbepoetin alfa infusion

**DOI:** 10.1186/s12348-015-0061-0

**Published:** 2015-10-26

**Authors:** Jonathan Li, Stephen E. Orlin, Karen E. Revere, John H. Kempen

**Affiliations:** Department of Ophthalmology, Scheie Eye Institute, 51 N 39th St, Philadelphia, PA 19104 USA; Department of Biostatistics and Epidemiology, Philadelphia, PA USA; Center for Clinical Epidemiology and Biostatistics, the Perelman School of Medicine, University of Pennsylvania, Philadelphia, PA USA

**Keywords:** Anterior uveitis, Iritis, Erythropoietin, Darbepoetin

## Abstract

A 79-year-old female with a 2-month history of newly diagnosed myelodysplastic syndrome for which she received blood transfusion with darbepoetin alfa presented with bilateral anterior uveitis 1 day after her fourth transfusion. On exam, visual acuity was 20/20 in both eyes with biomicroscopy notable for conjunctival injection and anterior chamber cell and flare consistent with anterior uveitis. She had no systemic symptoms, no history of eye trauma, and no known infections. This case, along with prior reports in the literature, suggests that anterior uveitis may be an idiosyncratic complication of darbepoetin alfa therapy.

## Findings

### Introduction

The proportion of uveitis cases attributed to drug-induced uveitis is reportedly less than 0.5 % [1]. Drug-induced uveitis more typically is caused by intraocular or topical rather than systemic medications [2]. However, systemic medications have become increasingly recognized as potential causes of uveitis. Several drugs are now more definitively associated with anterior uveitis, such as cidofovir, rifabutin, sulfonamides, bisphosphonates, fluoroquinolones, and diethylcarbamazine [3, 4]. Recognizing potential causes of medication-related intraocular inflammation is important because it can avoid unnecessary workups and may allow for clinical improvement by stopping the causative agent.

Although not well established, an association between epoetin alfa, a human recombinant erythropoietin, and anterior uveitis has been reported previously in the literature, with 13 patients across three medical centers developing anterior uveitis after receiving epoetin alfa during hemodialysis [2]. All of these patients were undergoing hemodialysis for end stage renal failure and receiving epoetin alfa for chronic anemia [2].

## Case report

A 79-year-old Caucasian female presented with acute onset, 1-day history of bilateral blurry vision, eye redness, photophobia, and tearing. Past medical history included a new diagnosis of myelodysplastic syndrome 2 months prior, for which she had received four blood transfusions with darbepoetin alfa, a longer-acting human recombinant erythropoietin. Her last transfusion with darbepoetin alfa was 1 day before presentation. She reported no other systemic symptoms, eye trauma, or underlying infections. She had no history of ocular inflammation or infection prior to beginning blood transfusions with darbepoetin alfa, which includes a 1-month period in which she had myelodysplastic syndrome but had not yet received any darbepoetin. She noted bloodshot eyes and ocular tenderness to palpation after two of her prior blood transfusions with darbepoetin alfa (to a degree that was less severe and which had spontaneously resolved).

On exam, her visual acuity was 20/20-2 in each eye. Her intraocular pressures were 15 mmHg in the right eye and 16 mmHg in the left eye. Anterior segment biomicroscopy revealed marked conjunctival injection, grade 2+ cell and flare in the anterior chamber, and 2+ nuclear sclerosis in each eye. There was no vitreous inflammation, and dilated fundus exam was normal without inflammatory signs.

She was diagnosed with bilateral, acute anterior uveitis and treated with prednisolone acetate 1 % hourly and homatropine 5 % three times a day. Two weeks later, she reported that all her symptoms had rapidly resolved; the visual acuity continued to be 20/20-2 in each eye and biomicroscopy demonstrated complete resolution of anterior uveitis.

Due to the temporal association of the patient’s darbepoetin alfa infusion with her acute onset anterior uveitis, and history of previous suggestive similar episodes just after infusions, a consensus decision was made with her hematologist to discontinue the use of darbepoetin alfa along with future blood product infusions. Treatment with decitabine, a DNA hypomethylating agent that leads to cellular differentiation, was initiated instead to help manage her myelodysplastic syndrome. The patient has since received two infusions of blood products without further episodes of ocular inflammation.

## Discussion

Our patient represents the first described case of likely darbepoetin alfa-induced anterior uveitis. It builds upon the previous reports of epoetin-induced anterior uveitis reported by Beiran et al. in 1996 [2].

The mechanism by which systemic medications induce ocular inflammation is largely unknown, with the exception of bisphosphonates, which are associated with increased intraocular inflammatory cytokines, elevated C-reactive protein, interleukin-1, and interleukin-6 [5]. Beiran et al. postulated a mechanism whereby erythropoietin could induce an inflammatory effect by altering prostaglandin E levels, breaking down the tight junctions that form the blood-aqueous barrier [2]. Darbepoetin alfa and epoetin alfa both have the same native protein sequence, differing only in glycosylation patterns [6]. Therefore, it is likely that similar mechanisms contributed to the drug-induced anterior uveitis in these previously reported cases as well as our own.

Given our patient’s underlying myelodysplastic syndrome—which can be associated with the development of autoimmune conditions—it also is possible that an autoimmune process was the cause of her anterior uveitis [7]. However, her reported history of two episodes of photophobia and eye redness after previous infusions, coupled with the absence of episodes both before beginning and after discontinuation of darbepoetin alfa infusions, suggests that this was indeed a drug-related anterior uveitis. Further studies are needed to elucidate the mechanism and strength between the association of darbepoetin alfa and anterior uveitis.

### Consent to publish

Consent to publish this case report has been obtained from the patient.
